# Naphthol as an Antiseptic Medicament

**Published:** 1888-02

**Authors:** 


### *NAPHTHOL AS AN ANTISEPTIC MEDICAMENT.*

Bouchard has communicated, to the French Academy of Sciences, the results of a series of researches, from which it appears that naphthol, one of the derivatives of naphthaline, is an excellent antiseptic, surpassing even iodoform.

Kaposi, several years ago, called attention to the value of this agent in the treatment of certain diseases of the skin, and particularly of scabies. He employs naphthol in a ten per cent. alcoholic solution, or in the form of ointment, and says that one or two applications will cure the most inveterate cases of common itch. For this purpose, an ointment may be made with one part of naphthol and ten of vaseline. He has had equal success with naphthol in psoriasis, eczema, and ichthyosis, and there is nothing better, he says, to allay tormenting itching of prurigo.

Heusinger also speaks in high praise of naphthol as an external agent (alcohol lotion or pomade) in the skin diseases mentioned above, in lupus erythematosus, and in chancres. Under its use, sores of a phagedemic character rapidly take in a healthy action. Van Harlingen, moreover, has seen favorable results from naphthol in obstinate cutaneous affections.

The experiments to which allusion has been made, have led Bouchard to regard naphthol as one of the best and safest of antiseptics. According to these, a three per cent. solution markedly retards the development of the typhoid bacillus, as well as that of the bacillus tuberculosis. The same solution is fatal to the microbes of several of the parasitic diseases of animals, and prevents fermentations. Bouchard gives naphthol internally in typhoid fever, and believes that, if it does not abort this disease, it certainly renders its course milder. He regards a dose of forty-five grains (2.50 grammes) a day as realizing the conditions of intestinal antisepsis, and affirms that, in such doses, there is no antiseptic agent which is more innocuous. This use of naphthol in typhoid fever has been followed to some extent in this country with apparently favorable results.—*Boston Medical and Surgical Journal.*

